# First record of *Lipeurus caponis* (Psocodea: Ischnocera: Philopteridae) parasitizing domestic chickens (*Gallus gallus domesticus*) in Brazilian Amazon

**DOI:** 10.1590/S1984-29612025040

**Published:** 2025-09-15

**Authors:** José Vicente Ferreira, Marcelo Cutrim Moreira de Castro, Alexandre Levi Monteiro Santana, Ahana Maitra, Felipe Arley Costa Pessoa

**Affiliations:** 1 Fiocruz Amazônia, Instituto Leônidas e Maria Deane – ILMD, Programa de Pós-graduação em Biologia da Interação Patógeno-Hospedeiro, Manaus, AM, Brasil; 2 Fiocruz Amazônia, Instituto Leônidas e Maria Deane – ILMD, Laboratório Ecologia de Doenças Transmissíveis na Amazônia, Manaus, AM, Brasil; 3 Instituto Nacional de Pesquisas da Amazônia – INPA, Laboratório de Entomologia Sistemática Urbana e Forense, Manaus, AM, Brasil; 4 Escola Superior Batista do Amazonas – ESBAM, Manaus, AM, Brasil; 5 Università degli Studi di Bari “A. Moro”, Dipartimento di Bioscienze, Biotecnologie e Ambiente Via Orabona, Bari, Italia

**Keywords:** Lice, Amazonas, colonial system, poultry production, Piolho, Amazonas, sistema colonial, produção avícola

## Abstract

Lice are obligatory ectoparasites of birds and mammals, possessing mouthparts adapted for feeding on the blood and/or keratinized tissue of their hosts. Recording parasites that may cause economic and sanitary losses in the country's poultry industry is of utmost importance. For this reason, in the present study, we report the first record of *Lipeurus caponis* (Linnaeus, 1758) parasitizing domestic chickens in the Brazilian Amazon, specifically in the municipality of Autazes, state of Amazonas. The insect was collected using entomological forceps and deposited in a 1.5 ml microtube containing absolute ethanol on a rural property located on the banks of the Paraná Madeirinha River, where chickens are raised in a colonial system, coexisting with animals of different species and fed a diet based on whole corn grains. A male specimen of *L. caponis* was collected from the interscapular tract during the rainy season. Given this finding, we highlight the importance of entomological surveillance to prevent potential epizootic outbreaks and mitigate impacts on regional poultry production.

## Resumo

Piolhos são ectoparasitos obrigatórios de aves e mamíferos, possuindo peças bucais adaptadas para se alimentar de sangue e/ou tecido córneo de seus hospedeiros. O registro de parasitos que podem ocasionar prejuízos econômicos e sanitários na avicultura do país é de suma importância. Por este motivo, no presente estudo relatamos o primeiro registro de *Lipeurus caponis* (Linnaeus, 1758) parasitando galinha doméstica na Amazônia brasileira, especificamente no município de Autazes, estado do Amazonas. O inseto foi coletado com auxílio de pinça entomológica e depositado dentro de um microtubo de 1,5 mL contendo etanol absoluto, em uma propriedade rural, localizada às margens do Rio Paraná Madeirinha, onde as galinhas são criadas em sistema colonial, convivendo com animais de diferentes espécies e com dieta a base de grão de milho inteiro. Foi coletado um espécime macho de *L. caponis*, na região do trato interscapular, durante época chuvosa. Diante desse achado, ressaltamos a importância da vigilância entomológica para prevenir possíveis surtos epizoóticos e mitigar impactos na produção avícola da região.

Lice are obligate ectoparasitic insects of birds and mammals belonging to the infraorder Phthiraptera, ranging from 0.3 to 11 mm in length (Linardi, 2024). They have a sclerotic body, flattened dorsoventrally, covered with backward-facing bristles, in addition to mouthparts adapted to feed on the blood and/or horny tissues of their hosts, with the majority being a permanent parasite, completing their entire biological cycle on the host, unable to survive outside it (Johnson & Clayton, 2003).

They were traditionally considered a distinct order, subdivided into two suborders, Mallophaga and Anoplura, based on their age, ecological, feeding habits and host interactions (Mjöberg, 1910). However, recent phylogenetic analyses have reclassified Phthiraptera as an infraorder within the order Psocodea, comprising four parvorders: Amblycera, Ischnocera, Rhynchophthirina (these three infraorders previously included in Mallophaga) and Anoplura (Johnson et al., 2018).

Representatives of Ischnocera are characterized by a head wider than the thorax and chewing-type mouthparts. These ectoparasites feed on desquamated cells from the epidermis and feathers, which can reach the dermis, eventually ingesting host blood as a food supplement. Their activity on the host can lead to intense pruritus, skin abrasions, and thermoregulatory disturbances due to feather loss (Johnson & Clayton, 2003).

Almost 3,910 louse species have been documented, capable of parasitizing avian hosts across all bird orders (Linardi, 2024). Among domestic chickens (*Gallus gallus domesticus* (Linnaeus, 1758)), the most frequently observed chewing lice include *Goniodes dissimilis* Denny, 1842, *Goniocotes gallinae* (DeGeer, 1778), in addition to *Menacanthus stramineus* (Nitzsch, 1818), and *Menopon gallinae* (Linnaeus, 1758), which have already been implicated as potential vectors of filarial worms and *Pasteurella multocida* (Chaves-Hernandez, 2014)

Another species that stands out is *Lipeurus caponis* (Linnaeus, 1758), which belongs to Ischnocera and is found both in colonial breeding systems and in industrial poultry farming, in addition to having been recorded in domestic pigeons (*Columba livia* Gmelin, 1789) (Naupay et al., 2015). It exhibits a cosmopolitan distribution and has been reported in several Brazilian states, including Alagoas, Bahia, Paraná, Pernambuco (Figueiredo et al., 1993), and São Paulo (Vaz, 1935; Figueiredo et al., 1993), as well as Ceará (Bastos, 2004), Maranhão (Guerra et al., 2008), Rio Grande do Sul (Freire, 1958, 1990; Santos et al., 2013), Paraíba (Santos et al., 2011), Rio de Janeiro (Amaral et al., 2007), Rio Grande do Norte (Fonseca et al., 2009; Ferreira et al., 2010) and Minas Gerais (Santos-Prezoto et al., 2003).

The occurrence of these ectoparasites in poultry houses is significantly influenced by several factors, including ground- or litter-based rearing systems, high bird population densities lacking adequate sanitary management, and the age of the birds, particularly between 36 and 72 weeks (Nadeem et al., 2007; Rezende et al., 2015).

Severe infestations can cause weight loss, decrease egg production, and reduce reproductive performance in poultry (Nadeem et al., 2007). These effects directly threaten Brazil's poultry production, considering that Brazil is the world's leading exporter and the second-largest producer of chicken meat, with an estimated flock of 250 million birds; the primary producers are the states of São Paulo, Paraná and Rio Grande do Sul (Brasil, 2023a; ABPA, 2023).

The state of Amazonas ranks 13th nationally in poultry production, with a total flock exceeding 3 million birds (Brasil, 2023a). The metropolitan region of Manaus is the largest producer in the state, with emphasis on the municipalities of Manaus (1,600,000 birds), Manacapuru (326,000), and Iranduba (297,526) (Brasil, 2023a). Moreover, according to data from IBGE (Brasil, 2023a), the municipality of Autazes, located approximately 113 km from the state capital, maintains a flock of 9,167 birds and stands out as an important producer in family farming (Amazonas, 2011).

The municipality of Autazes, dairy, and family farming constitute the primary economic activities. Although poultry farming has a limited commercial presence, it remains an essential component of household subsistence for families engaged in smallscale bird rearing. No poultry farms are officially registered in the region, according to data from the Amazonas Agricultural and Forestry Defense Agency (Amazonas, 2011, 2024; Cruz et al., 2016).

Given the potential economic and health impacts of parasitic infestations in poultry farming, particularly in regions where data are limited, it is essential to document the occurrence of such parasites. This is especially pertinent in northern Brazil, where research remains scarce, and understanding the geographic distribution of these organisms is critical. Accordingly, the present study aims to report the first occurrence of *L. caponis* in domestic chickens in Autazes, Amazonas State, Brazil.

The municipality of Autazes, located in the state of Amazonas, Brazil, is characterized by a humid tropical climate (Köppen classification: Af), several rivers and lakes, and an average elevation of approximately 23 m above sea level, with temperatures typically ranging from 24°C to 34°C and year-round precipitation (WeatherSpark, 2024).

**Figure 1:**
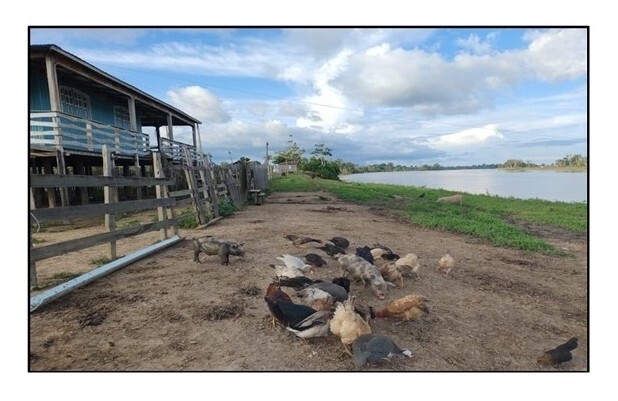
Animals being fed whole corn grain around the property headquarters, located in a floodplain area.

Fieldwork was conducted on a rural property (03º50'59” S 59º39'28” W) located on the banks of the Paraná Madeirinha River. The property encompasses various ecosystems, including floodplain, igapó (blackwater) forests, and dense terra firme (dryland) tropical forest. Birds were reared in a traditional colonial system in facilities close to the property's main residence, with a diet based on whole corn grains and natural pasture. The birds shared the environment with other domesticated species, such as cattle (*Bos taurus* (Linnaeus, 1758)), buffalo (*Bubalus bubalis* (Linnaeus, 1758)), pigs (*Sus scrofa domesticus* (Linnaeus, 1758)), ducks (*Cairina moschata domestica* (Linnaeus, 1758)), and guinea fowl (*Numida meleagris* (Linnaeus, 1758)) (Figure 1).

The louse specimen was extracted using entomological tweezers and immediately transferred to a duly labeled 1.5 ml microtube containing absolute ethanol for preservation. The specimen was processed without clarification, mounted on a slide in Canada balsam, and the taxonomic identification made with specific taxonomic key (Tuff, 1977). The images were captured with a Leica DFC500 digital camera coupled to a Leica M205c stereoscopic microscope, connected to a computer with the Leica Application Suite (LAS) V3.6 software, which includes a selfassembly module. The specimen was deposited in the Laboratório de Entomologia of the Instituto Leônidas e Maria Deane | Fiocruz Amazônia.

**Figure 2:**
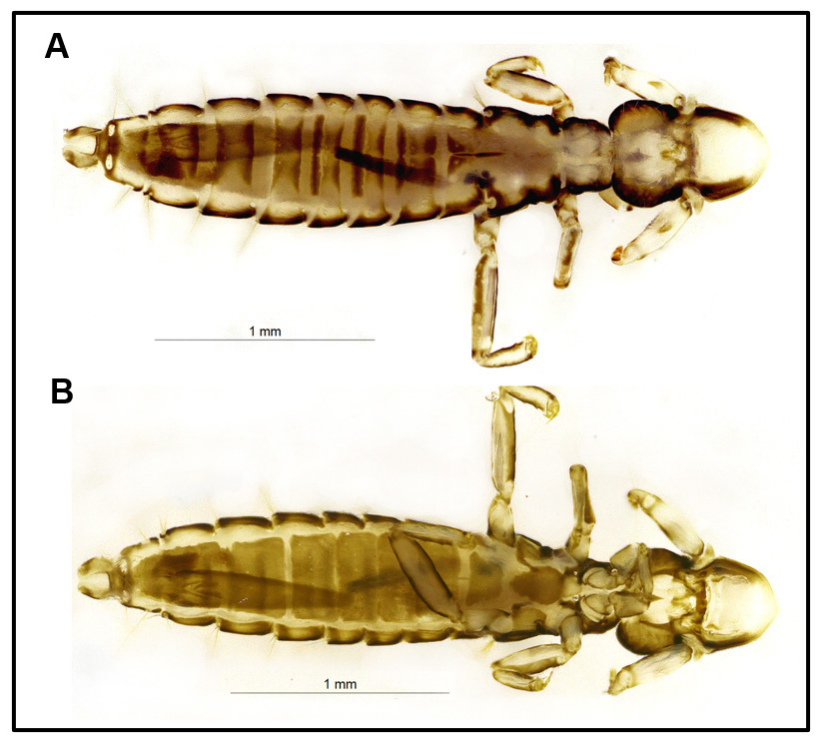
*Lipeurus caponis* Male. Dorsal View (A). Ventral view (B).

The specimen, identified as a male of *L. caponis* (Figure 2), was collected from the interscapular feather tract of a 39-week-old domestic chicken during the rainy season in January 2022.

The species is morphologically characterized by an elongated and narrow body, approximately 2.5 mm in length and 0.3 mm in width, including narrow legs, with the posterior pair being twice the length of the anterior. Additionally, a distinguishing angular projection is present anterior to the antennae. Sexual dimorphism is evident; males possess an elongated first abdominal segment and a thickened abdominal appendix (Johnson & Clayton, 2003). It can be differentiated from other species of the same genus by its brown head on the lateral margin, bi-segmented antennae, sickle-shaped tarsal claws, mesosternum with a circular brown shield, metasternum with a trapezoidal brown shield, dorsal posterior margin of the pterothorax without a group of setae, lateral posterior margin of the pterothorax with long setae and the terminal abdominal tergite with two dorsolateral depigmented punctuations (Tuff, 1977).

**Figure 3:**
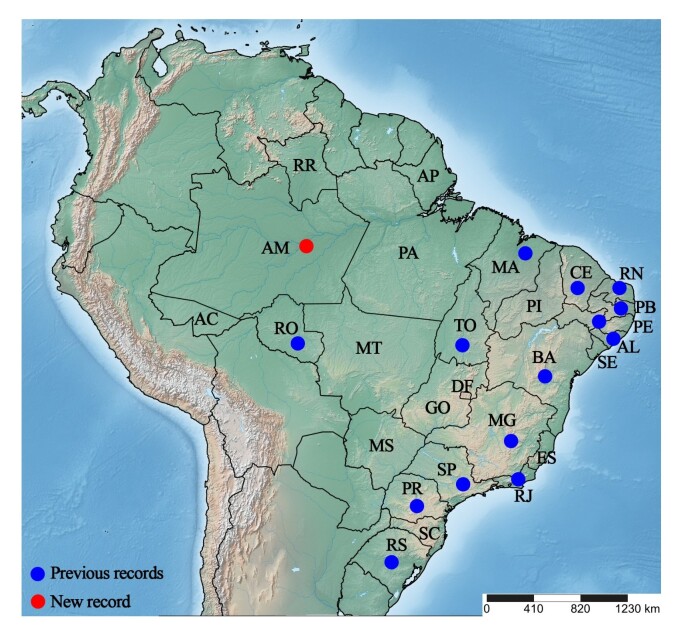
Distribution of *Lipeurus caponis* in Brazil, identifying the previous records (blue dots) and the new record (red dot).

Over the past five years, poultry farming in the northern Brazil, especially in Amazonas, which emerged in 2023 as the main producer of chickens and eggs in the region, has shown significant advances in nutrition, health control, breeding and overall farm management practices. These improvements are reflected in notable increases in productivity, largely driven by research activities aimed at the development of this sector such as the present study, which reports the first documented occurrence of this louse species in the Brazilian Amazon (Figure 3). Although relatively common in other high poultryproducing areas of Brazil, its presence duly recorded in the present study in Brazilian Amazon highlights the growing importance and intensification of poultry farming in this part of the country (Brasil, 2023b).

Lice remain a neglected group of ectoparasites, with few specialists and this study represents the first investigation lice in domestic chickens in the Brazilian Amazon, northern Brazil, state of Amazonas. These parasites are capable of causing epizootic outbreaks and can also facilitate the introduction of novel pathogens, highlighting the need for systematic entomological monitoring and further research to understand their role in animal health and their impact on epidemic and poultry farming.

## Data Availability

The data used in the study are publicly available
